# Dynamic Neurocognitive Adaptation in aging: developing and validation of a new scale

**DOI:** 10.1002/alz.087584

**Published:** 2025-01-09

**Authors:** Filippo Cieri, Giulia Di Francesco, Andrew R. Bender, Chad L. Cross, Jessica ZK Caldwell

**Affiliations:** ^1^ Cleveland Clinic, Las Vegas, NV USA; ^2^ Pescara Hospital, Pescara, Pescara Italy; ^3^ Cleveland Clinic Lou Ruvo Center for Brain Health, Las Vegas, NV USA; ^4^ University of Nevada Las Vegas, Las Vegas, NV USA

## Abstract

**Background:**

Up to 40% of current AD cases may have been preventable through protective factors, such as physical and cognitive activities, preventing neurodegeneration. Reserve, resilience, and resistance are key concepts in cognitive neuroscience of aging, and they share common adaptive mechanisms. In this paper we propose a development and validation of a new scale, called dynamic Neurocognitive Adaptation (dNA).

**Method:**

This study included 815 participants (50% women) with a minimum age of 65 years, divided in two subsamples: exploratory factor analysis (EFA), and confirmatory factor analysis (CFA). Data collection utilized survey measures administered via *Qualtrics*. Our initial scale was composed of 30 items, investigating 7 different dimensions, explored by a 5‐point Likert scale reflecting frequency of activities. We investigated lifetime through 7 time‐windows, from childhood (0‐10) to old age (65+).

**Result:**

Our final scale is composed by 20 items divided among 4 dimensions (physical, cognitive, social, and creative). Scale assessment indicated that there were no issues found related to multi‐collinearity (*r*< 0.95) or non‐collinearity (*r*< 0.3). For the final model, KMO = .80 and Bartlett’s test of sphericity indicated all values ≤0.01. Our Cronbach’s alpha was .83.

**Conclusion:**

We have validated a reliable, novel, easy to complete, and comprehensive scale to assess lifetime adaptive behaviors, which can be applied in research on AD risk reduction, MCI, and in clinical practice. We underlined the dynamic role of time and the investigation of the creative dimension in aging. Our dNA can be used to personalize health recommendations in aging.

